# Supplementary material for: Patterns of Intron Gain and Loss in Fungi
Source: PLoS Biol. 2004 Nov 30;2(12):e422. doi: 10.1371/journal.pbio.0020422 (PMC532390; doi:10.1371/journal.pbio.0020422)
Supplement: Table S1 — Also available at http://genes.mit.edu/NielsenEtAl/. (4.3 MB ZIP). [file pbio.0020422.st001.zip › NielsenEtAl/html/972.html]

AN3144.1.NCU07584.1.MG09837.1.FG04221.1


```
 CLUSTAL W (1.82) Multiple Sequence Alignments - Introns Inserted


Sequence 1: MG09837.1	743 aa
Sequence 2: FG04221.1	674 aa
Sequence 3: NCU07584.1	674 aa
Sequence 4: AN3144.1	675 aa
Alignment Length: 758 aa
Number Identitical Residues: 246 aa
Alignment Score (without introns) 13154


MG09837.1 	MSGRKLGGGRILGSGKGLAPPAPPPPQPQQHQHQH----HGSVRAPSPFAASEVSSISSG
NCU07584.1	MSGRKLGGGRILGNGKGLAPPTAAAAAAASSSSPSPSTIAAIHRAASPYAPSDSSTVSFI
FG04221.1 	MSGRKLGGGRVLGSGRGLAPPTPPVTN--------------APRVASPLAASE-SSISIA
AN3144.1  	MSRRTLGGGRILGSANTRTTATLSSPQ---------------IRPNNILSPSA-STLSLN
          	** *.*****:**...  :..: . .                 *  .  :.*  *::*  

MG09837.1 	SHN--QNTITPPTSSSPLPDFSQDLAANISLDAPSPSNAGAGGL-----VCPICQEEM0L
NCU07584.1	SPNSTRESISP-SSSAPLPHIPQDLVSNVSLPGPSNGGAQSVGANDNRLVCPICQEEM~V
FG04221.1 	SS-----SISPPISGISPDLAGQDIGNSISVGAQGKGGAADGG-------------AL0M
AN3144.1  	SQ----------ASASQLSSETQDLTSRISLDNGSAPLPAAPAATGAQLSCPICSEEM0V
          	*            *.       **:   :*:   .   .   . ..    .. ... : :

MG09837.1 	TLLQLNR~HIDDMHQELPQVQHDEVMTWIDKQVLKAKKLQPLSLLSQKLRGLEVFESNE-
NCU07584.1	TLLQLNR~HIDDVHQELPEETQDEVKSWFDKQVRKAKKFQPLNIINQTLRGLDVFESNET
FG04221.1 	TLLQLNR~HIDDTHQELPEEEQDEVKTWFDKQVLKAKRFQPLSLINQKLRGLDVFESNE-
AN3144.1  	TLLQLNR2HLDDVHHNLEDDRQDEVKDWFKTQMEKAKRFQPLAVLNQKLKGLDVFESNEN
          	******* *:** *::* :  :***  *:..*: ***::*** ::.*.*:**:******.

MG09837.1 	DPIINNNVQSSAHHSSSSNATVPRVVEPSVIDPDDLITKKHWQRSTGGDSCTELTCDRRL
NCU07584.1	QPVLATSAIATAPGRPAEKA----------VDPEELVTRHHWQRPTGNDPCTDPTCTRKL
FG04221.1 	SVPVAPPISTVAGKNPLEGP----------IDPDELITRHHWQRSTSYDLCTDPACGRSL
AN3144.1  	HQALTGASRPSGLSQNHQSQSFLHEAQPRPPDPEEIITKEHWQPRGFYDTCLEPSCGQRL
          	   :     . .     .  :     ..   **::::*:.***     * * : :* : *

MG09837.1 	GPLSGSINCRKCGLLFCEEHTMYQMRLSPRTATHDPARGHWYRVCETCYKSREGYNDHNG
NCU07584.1	GPFSGLVNCRKCGRLFCEEHTMYQMKLS-RSANHEPVRGIWCRVCETCFKSREGYNDHWG
FG04221.1 	GPLNGSINCRHCGLLFCEEHTMYQMKLS-RSAKHEPVRGYWARVCETCFKSREGYNDHQG
AN3144.1  	NATNGCVNCRKCGKLFCEEHTMYQMKLS-RSAQHEPVRGLWYRVCETCYKSREGYNDHNG
          	.. .* :***:** ***********:** *:* *:*.** * ******:********* *

MG09837.1 	~LITDHTAAFVAIRKQRVERQNLDIARLEKRLTKLTQLLASPLEEVAAMTPGAGGLLTSP
NCU07584.1	~TVRDHMAEFKAIRAKRVDRHKLEVQRLEKRLTKLTRLLAEMPPEE-----------GSG
FG04221.1 	~VLTDHTNAFVEIRRKKVERQNLEISRLEKRLTKLTKLLANPPENLTQSN----GSLLGP
AN3144.1  	1LVRDRMDEFKAIRKPIVDKSLLEVSRLEKRLTRLTQLLASLSPEQLQ---------SNK
          	  : *:   *  **   *::  *:: *******:**:***.   :             . 

MG09837.1 	IATLAGQKSTRKAIEQSVVTWEDDASVARCPFCQQEFGSWTFRRHHCRICGRVVCADPQT
NCU07584.1	LLALTGQKSQRKMIEQSVVSWEEDGAVTNCPFCKQEFGSWTFRRHHCRICGKVVCADPET
FG04221.1 	VTSLAGQKNPRKLIEQSVVTWEEDATVSKCPFCQQEFGSWTFRRHHCRICGRVVCGDPQT
AN3144.1  	LWPIPFQHDERKALEQTVVSWQDDTSVTRCPFCQQDFSSYTFRRHHCRTCGRVVCGDVLT
          	: .:. *:. ** :**:**:*::* :*:.****:*:*.*:******** **:***.*  *

MG09837.1 	GC~SAEIGLNVASVPQRPSLLQQNSSTYSSTSLGPLRSTAL~EKRLDPGAGSQGQISVSV
NCU07584.1	AS1-----------------------------------TIN~TEKPPRIVTTT----LDI
FG04221.1 	AC~SSEVGLNVS-----------------SDTNGATKSHPG~TEKPQP-ATNGGQVGIDI
AN3144.1  	GC~SVEVPLS---------------------------VTPL1SKAYLEKSVNVGSLNIDI
          	.. : .   .                                 :       . .. .:.:

MG09837.1 	RMCRDCSTTIFSKRDVAASIAYKPADQRAYENLRQFERGIRSLMPSFQRSLLALQ~PPDP
NCU07584.1	RMCRDCKSTIFSHRDFAASIAHKPPDQRAYETLRQFERGIRMLMPSFQKALLALQ~PPNE
FG04221.1 	RMCRDCKHTIFSARDFAASLQHKPADQRAYETLQQFERGIQQLLPSFHRVLLNLQ~PEKK
AN3144.1  	RLCKECKSTLFDRSDFEADVARKPADVRAYENLRQFERGIRLLMPKFQKLLTTLQ2NP--
          	*:*::*. *:*.  *. *.:  **.* ****.*:******: *:*.*:: *  **     

MG09837.1 	NN-LHALEKPPPTHAQIQEAAKIRKRLMDAFAKYNLAAKRLRDLPTDNPAQKKLQLAVHA
NCU07584.1	DD--YRQDKPPPTSAQVQEAAKIRKRLTDAFAKYDLAARRLRDMKTDSPTQLRLQQSVYA
FG04221.1 	ESGEIDLNKPPPTRAQIQEAAKIRKRLVDSFGKYGTAAKRLRDLPTESATQRRLQAAIYT
AN3144.1  	--------RRPPSAAQLAEASKVRKRLTDSFTQYDTAARRIRDLPTNSTTQQRLQKAIYQ
          	        : **: **: **:*:**** *:* :*. **:*:**: *:..:* :** ::: 

MG09837.1 	AASTFLHTHMLPLKSVPHMLRSSSSSKPSSAGQQHRRMLSGLNHGNGLAGHLSPLRNGET
NCU07584.1	AASAFLHANLIPLKSVPRVLRS------NSMQHQHRRLLNGSTSTL------SPLRNGES
FG04221.1 	YASGFLHTNMLPLKSLPQLLRSRSSAS-SSTAISASRLLASHNHSG------SSLRHSE-
AN3144.1  	QATNFLHLHMLPLKTLPKILKHATPDGSQPSSSSSPDSPGGASVEG-HRRQNSALAAVKS
          	 *: *** :::***::*::*:  :.  ...   .      . .         *.*   ::

MG09837.1 	-LAGDGDASSVAGS-EVSTAASALETEEKELRERLVVLEEQRFLVQDMVNNARGARRFEE
NCU07584.1	AFSKDAETSSNAGASEASVVQSELDLEEKEAKERLVVLEEQMFMVQEMLKSARAQRRFEE
FG04221.1 	--LADTETSSQAPS-EGSTVVSQLETEEKELRERLVVLEEQRFMVESMIKTAHGSRRFEE
AN3144.1  	GKVAASISNSSIAS-EASSAVSALEAEEKALRERLIVLEEQKFFVSEMIADANRRRKFDE
          	.      :.*   : * * . * *: ***  :***:***** *:*..*:  *.  *:*:*

MG09837.1 	VGALSGNLEELDREIATLKRQVAGVEEKWEGLYATGQGTPGAAR
NCU07584.1	VSALNRNVEELEAEIERAKEGVRGVEERMGALYIGG--------
FG04221.1 	VSALSRNVDELDAEINELKNKVGGIEERWEGVYRNGVT------
AN3144.1  	VSSLAMNIEDLSREIDRVNGLLDGLD--FEGAYMGNHT------
          	*.:*  *:::*. **   :  : *::    . *  .
```
